# Analysis of the StoRM cohort reveals physical activity to be associated with survival in metastatic breast cancer

**DOI:** 10.1038/s41598-020-67431-6

**Published:** 2020-07-01

**Authors:** Lidia Delrieu, Emmanuelle Jacquet, Céline Segura-Ferlay, Ellen Blanc, Olivia Febvey-Combes, Christine Friedenreich, Gilles Romieu, William Jacot, Maria Rios, Pierre-Etienne Heudel, Célia Roemer-Becuwe, Christelle Jouannaud, Olivier Tredan, Loïc Chaigneau, Monica Arnedos, Hubert Orfeuvre, Nathalie Quenel-Tueux, Jean-Philippe Jacquin, Jean-Marc Ferrero, Isabelle Moullet, Sophie Abadie-Lacourtoisie, Frédérique Penault-Llorca, David Cox, Thomas Bachelot

**Affiliations:** 1 Laboratory of Motor Biology (LIBM), EA7424, Vascular Biology and Red Blood Cell Team, Claude Bernard Lyon 1 University, Villeurbanne, France; 2 Cancer and Environment Department, Centre Léon Bérard, Lyon, France; 3 Oncology and Blood Diseases Department, Joseph Fourier University, University Hospital Center, Grenoble, France; 4 Direction of Clinical Research and Innovation (DRCI), Centre Léon Bérard, Lyon, France; 5 Department of Cancer Epidemiology and Prevention Research, CancerControl Alberta, Alberta Health Services, Calgary, AB Canada; 6 Departments of Oncology and Community Health Sciences, Cumming School of Medicine, University of Calgary, Calgary, AB Canada; 7 Institut du Cancer de Montpellier (ICM) Val d’Aurelle, Montpellier, France; 8 Department of Medical Oncology, Cancer Institute of Lorraine - Alexis Vautrin, Vandoeuvre Les Nancy Cedex, France; 9 Oncology Department, Centre Léon Bérard, Lyon, France; 10 Oncology Center of Gentilly, Nancy, France; 11 Medical Oncology Service, Jean Godinot Institute, Reims, France; 12 Medical Oncology Service, University Regional Hospital Center, Besançon, France; 13 Department of Medical Oncology, Gustave Roussy Cancer Campus, Villejuif, France; 14 Medical Oncology Service, Fleyriat Hospital Center, Bourg en Bresse, France; 15 Medical Oncology Department, Bergonié Institute, Bordeaux, France; 16 Medical Oncology Department, Lucien Neuwirth Oncology Institute, Saint Priest en Jarez, France; 17 Medical Oncology Department, Antoine Lacassagne Center, Nice, France; 18 Medical Oncology Department, Sauvegarde Clinic, Lyon, France; 19 Western Cancer Institute, Paul Papin site, Angers, France; 20 Jean Perrin Center, and UMR INSERM 1240 IMoST, Clermont-Ferrand, France; 21 Cancer Research Center of Lyon, INSERM U1052, Centre Léon Berard, Lyon, France

**Keywords:** Prognosis, Breast cancer

## Abstract

Benefits of physical activity are widely demonstrated for early stage cancers but few studies have focused on metastatic disease. The purpose of this study was to determine the impact of physical activity on survival in patients with metastatic breast cancer. We conducted a secondary analysis of the national, multicentric, non-randomized, prospective cohort SNPs to Risk of Metastasis (StoRM) study. The level of physical activity was self-reported at inclusion and divided into three categories of physical activity: light level, moderate level, and vigorous level. Overall, 833 patients (56.2%) completed the physical activity questionnaire at baseline on average physical activity during the previous year: 11.6% had a light level of physical activity, 69.0% achieved moderate levels of physical activity and 19.3% reported vigorous levels of physical activity. After adjustment for confounding, physical activity was not statistically significantly associated with overall survival in the whole population. Subgroup analysis identified that both vigorous and moderate physical activity were associated with statistically significantly improved overall survival compared to light physical activity level only in the HER2 positive subgroup (HR 0.23; 95% CI 0.07–0.70, *p* = 0.01 and HR 0.38; 95% CI 0.15–0.96, *p* = 0.04). Physical activity done during the previous year was associated with survival in HER2 positive metastatic breast cancer patients. These results suggest that overall survival in metastatic breast cancer patients could be improved through physical activity which should be considered as a complementary intervention for these individuals. The study showed that moderate/vigorous levels of physical activity were associated with better overall survival, and that these associations remained statistically significant in multivariate analysis in the HER2 positive subgroup. These results have clinical relevance and justify the recommendations for physical activity interventions in metastatic breast cancer.

## Introduction

Breast cancer is the main cause of cancer death among women worldwide with more than 1.6 million new cases diagnosed annually and 58,968 cases estimated in France in 2017^1^. About 5% of breast cancers are metastatic at diagnosis and 20–30% of localized breast cancers become secondarily metastatic. Once metastases are detected, metastatic breast cancer is considered an incurable disease, and the primary aim of treatment is to increase survival without excessive toxicity and symptomatic control^2–5^. Although in recent years new therapies in metastatic breast cancer have improved life expectancy of these patients, median overall survival is limited to 3–4 years and only 25–30% of the patients survive five-years or more^6–10^. Survival appears to be closely linked to non-modifiable clinical and biological prognostic factors, which may influence therapeutic strategy. Some of these factors are now well described in the literature: performance status, modified Bloom–Richardson–Elston grading, site and number of metastases, hormone receptor status, Her-2 status, disease free-interval and previous adjuvant chemotherapy^6,11–14^.

Clinical benefit of physical activity has been widely demonstrated for early stage of breast cancer^15–19^. Currently, few studies have examined the impact of physical activity on patients with metastatic breast cancer, and only five intervention studies worldwide focused on the implementation of physical activity in this population^20–24^. Most intervention studies analyzed the benefits of physical activity in terms of quality of life and reduction of symptoms, and only one study assessed the impact of physical activity on survival among a small number of pre-selected patients^25^. The need, desire and ability of these patients to engage in physical activity programs have been reported^21,24,26,27^. The purpose of this secondary analysis is to determine the impact of physical activity on the survival of patients with metastatic breast cancer in the three different histological subgroups (human epidermal growth factor receptor-2 (HER2) positive, estrogen receptors (ER)+ or progesterone receptors (PR)+ and HER2 negative and triple negative (HER2−/ER−/PR−)).

## Methods

### SNPs to risk of metastasis (StoRM) cohort

StoRM is a national, multicentric, prospective cohort study of metastatic breast cancer patients, designed to identify genetic and other factors associated with metastatic relapse and survival. The main inclusion criteria were: women/men aged 18 years or older, with a histologically proven breast cancer that was metastatic for less than one year, and with an immunohistological analysis from the primary tumor (ER and PR status, and HER2 status). Exclusion criteria were other coexisting cancer, or another cancer diagnosed within the last 5 years, and the inability to undergo medical monitoring for geographical, social or psychological reasons.

From March 2012 to May 2014, 1,483 patients were included, time to progression on the first metastatic treatment was recorded, they were followed until death, every six months for three years, and then annually until July 2017.

We report here a secondary analysis of patients enrolled in the StoRM cohort who completed a physical activity questionnaire administered at baseline on average physical activity during the previous year.

The study was conducted in accordance with the International Conference on Harmonization Good Clinical Practice standards and the Declaration of Helsinki. Patients provided written informed consent; the study was approved by the relevant institutional review board (South-East IV Patient Protection Committee, 26 October 2011, No.: 11/089).

### Assessment of physical activity

The level of physical activity was self-reported at the time of enrollment into the cohort study using a questionnaire that 11 items, derived from a modified version of the Baecke questionnaire and already used in a French cohort study^28,29^. Questions included the usual distance walked daily (< 500, [500–2,000], and ≥ 2,000 m), the average number of flights of stairs climbed daily (0, 1–4, and ≥ 5), the average amount of time (in hours) spent weekly doing light household activity (0, 1–4, ≥ 6) and heavy household activity (0, 1–4, ≥ 6), the average of time (in hours) spent weekly doing moderate recreational activity (0, 1–4, ≥ 6), and vigorous recreational activity (0, 1–4, ≥ 6). Questions were coded and converted in metabolic equivalent of task (MET) per minute and per week using the Compendium of Physical Activities^30,31^. We multiplied the MET corresponding to the energy expenditure during a specific physical activity by its frequency and duration. By using the Compendium of Physical Activities, we assigned the value of 7.5 METs for vigorous physical activity, 5 METs for moderate physical activity, 4 METs for heavy household, 3 METs for light household, 8 METs for walking up 120 floors and 3 METs for walking one hour. The global score of physical activity was divided into three categories commonly used by physical activity guidelines and by the World Health Organization (< 10 MET-hours/week is equivalent to light physical activity, between 10 and 50 MET-hours/week is equivalent to moderate physical activity, and over 50 corresponds to vigorous physical activity)^19^.

### Statistical analysis

Qualitative data were described by frequency and percentage and quantitative data were described by mean and standard deviation (SD). Comparisons were performed using χ^2^ test or Student’s t-test (ANOVA when more than 2 groups are compared) for qualitative and quantitative data, respectively. Overall survival was defined as the time from the date of the first metastasis until death due to any cause. Any patient not known to have died at the time of analysis was censored on the last recorded date on which the patient was known to be alive. Overall survival distribution was estimated using the Kaplan–Meier method and described in terms of median along with the associated two-sided 95% confidence interval. Distributions were compared between physical activity groups using a Log-Rank test. Overall survival according to physical activity analysis was made in the three different histological subgroups (HER2 positive, ER+ or PR+ and HER2 negative and triple negative). To counteract the problem of inflated type I errors due to multiple subgroup comparisons, Bonferroni correction has been used and defined a new two-sided alpha significance level of 0.0166 for these three subgroup analyses.

A multivariate analysis using Cox-regression models was performed to estimate the hazard ratios (HR) and their 95% confidence intervals [CIs] after adjustment for baseline characteristics that were potential confounders including age at metastatic diagnosis, body mass index, Eastern Cooperative Oncology Group (ECOG) Status, Performance status (PS), smokers, education, number of metastatic sites, adjuvant chemotherapy, metastatic at diagnosis and tumor type (Luminal-like, HER2+, Triple Negative). Several baseline characteristics had missing values, thus a multiple imputation approach was applied using the method developed by Rubin in order to integrate important variables such as PS into multivariate analysis^32,33^.

Subgroup analyses by tumor type (Luminal-like, HER2+, Triple Negative) were performed using the same methods as used for the whole population. Since dual HER2 inhibition has been shown to improve overall survival, dual HER2 inhibition (trastuzumab and pertuzumab/lapatinib versus trastuzumab) as first line treatment was added to the model for patients with HER2 positive metastatic breast cancer.

All statistical analyses were performed using SAS (version 9.4).

## Results

### Characteristics of the study population

Among 1,483 patients included in the StoRM cohort, 833 (56.2%) completed the physical activity questionnaire. Demographics and clinical characteristics of the study population (n = 833) are presented in Table 1. The mean age of women at metastatic diagnosis was 57.8 years (SD 13.1). Concerning anthropometric data, 31 patients (3.7%) were considered as underweight (body mass index [BMI] < 18.5), 401 women (48.4%) had a normal weight (BMI 18.5–25), 234 (28.2%) were overweight (BMI 25–30) and 163 (19.7%) were obese (BMI > 30). The majority of women (88.9%) had a performance status (PS) of 0 or 1 and 25% of breast cancers were metastatic at diagnosis. Among the 833 patients, 144 (17.3%) were HER2 positive, 568 (68.2%) were luminal (ER+ and/or PR+ and HER2 negative) and 119 (14.3%) were triple negative. Bone was the most common site of metastases (69%). The median for time between initial diagnosis of cancer and metastatic disease was 53.5 months (interquartile range: 0.1 to 462.5 months).

**Table 1:** Demographics and clinical characteristics of women with metastatic breast cancer depending on physical activity level.

	Total physical activity (MET-hours/week)			Total N = 833
	Light physical activity (< 10 MET-hour/week) N = 97 (12%)	Moderate physical activity ([10;50[ MET-hour/week) N = 575 (69%)	Vigorous physical activity (≥ 50 MET-hour/week) N = 161 (19%)	
**Demographic**				
Age at primitive tumor diagnosis				
N	97	573	161	
Mean ± sd	62.4 (± 12.0)	52.1 (± 12.8)	50.8 (± 11.1)	53.1 (± 12.8)
Age at metastatic diagnosis				
N	97	575	161	
Mean ± sd	67.2 (± 11.6)	56.9 (± 13.1)	55.7 (± 11.4)	57.8 (± 13.1)
Weight, kg				
N	97	572	161	830
Mean ± sd	69.5 (± 16.6)	68.3 (± 14.7)	64.6 (± 12.9)	67.7 (± 14.7)
Missing data	0	3	0	3
Body mass index, kg/m				
Underweight (< 18.5 kg/m^2^)	6 (6.2%)	18 (3.2%)	7 (4.3%)	31 (3.7%)
Normal weight (< 25 kg/m^2^)	36 (37.1%)	274 (48.0%)	91 (56.5%)	401 (48.4%)
Overweight (25–30 kg/m^2^)	27 (27.8%)	163 (28.5%)	44 (27.3%)	234 (28.2%)
Obese (> 30 kg/m^2^)	28 (28.9%)	116 (20.3%)	19 (11.8%)	163 (19.7%)
Missing data	0	4	0	4
PS ECOG				
0–1	55 (70.5%)	413 (90.8%)	128 (93.4%)	596 (89.0%)
2–3	23 (29.5%)	42 (9.2%)	9 (6.6%)	74 (11.0%)
Missing data	19	120	24	163
Metastatic at diagnosis				
No	54 (55.7%)	363 (63.5%)	104 (64.6%)	521 (62.8%)
Yes	31 (32.0%)	136 (23.8%)	37 (23.0%)	204 (24.6%)
Undetermined	12 (12.4%)	73 (12.8%)	20 (12.4%)	105 (12.7%)
Missing data	0	3	0	3
Education				
High School	64 (73.6%)	274 (51.1%)	65 (43.3%)	403 (52.1%)
1- to 2-year university degree	17 (19.5%)	156 (29.1%)	48 (32.0%)	221 (28.6%)
> 2-year university degree	6 (6.9%)	106 (19.8%)	37 (24.7%)	149 (19.3%)
Missing data	10	39	11	60
Smokers				
Yes	26 (27.4%)	255 (44.8%)	67 (41.6%)	348 (42.2%)
No	69 (72.6%)	314 (55.2%)	94 (58.4%)	477 (57.8%)
Missing data	2	6	0	8
**Clinical**				
Molecular subtype				
HER2 positive	12 (12.6%)	96 (16.7%)	36 (22.4%)	144 (17.3%)
Luminal	71 (74.7%)	394 (68.5%)	103 (64.0%)	568 (68.4%)
Triple negative	12 (12.6%)	85 (14.8%)	22 (13.7%)	119 (14.3%)
Missing data	2	0	0	2
Number of death	63	337	75	475
HER2 positive	8	47	8	63
Luminal	42	223	46	311
Triple negative	12	67	21	100
Median between diagnosis and metastatic disease (months)[range]	73.5 [0.7;335.5]	49.0 [0.1;462.5]	57.2 [0.3;322.5]	53.5 [0.1;462.5]
< 24 months	14 (14.4%)	133 (71.9%)	38 (20.5%)	185 (100%)
≥ 24 months	52 (53.6%)	306 (68.9%)	86 (19.4%)	444 (100%)
CA 15.3 [U/ml]				
N	76	449	118	643
Mean ± sd	338.9 (± 694.6)	212.1 (± 543.0)	198.8 (± 499.4)	224.7 (± 556.2)
Missing data	21	126	43	190
Lymphocytes [G/l]				
N	73	446	122	641
Mean ± sd	1.9 (± 1.9)	1.8 (± 1.1)	1.8 (± 1.2)	1.9 (± 1.2)
Missing data	24	129	39	192
Number of metastatic sites				
< 3 sites	60 (61.9%)	381 (66.3%)	120 (74.5%)	561 (67.3%)
≥ 3 sites	37 (38.1%)	194 (33.7%)	41 (25.5%)	272 (32.7%)
Metastatic localization				
Brain	8 (8.2%)	31 (5.4%)	6 (3.7%)	45 (5.4%)
Bones	70 (72.2%)	400 (69.6%)	105 (65.2%)	575 (69.0%)
Pulmonary	33 (34.0%)	152 (26.4%)	39 (24.2%)	224 (26.9%)
Node	31 (32.0%)	189 (32.9%)	57 (35.4%)	277 (33.3%)
Pleura	19 (19.6%)	59 (10.3%)	17 (10.6%)	95 (11.4%)
Skin	12 (12.4%)	35 (6.1%)	8 (5.0%)	55 (6.6%)
Hepatic	33 (34.0%)	202 (35.1%)	51 (31.7%)	286 (34.3%)
Loco regional	12 (12.4%)	109 (19.0%)	36 (22.4%)	157 (18.8%)
Others	10 (10.3%)	76 (13.2%)	19 (11.8%)	105 (12.6%)
Adjuvant chemotherapy				
Yes	38 (39.2%)	314 (54.6%)	86 (53.4%)	438 (52.6%)
No	59 (60.8%)	261 (45.4%)	75 (46.6%)	395 (47.4%)
Dual inhibition of HER2				
Yes	3 (27.3%)	32 (35.6%)	13 (39.4%)	86 (64.2%)
No	8 (72.7%)	58 (64.4%)	20 (60.6%)	48 (35.8%)
Missing data	1	6	3	10

### Physical activity level

Data on physical activity level are presented in Table 2. The mean MET-hours/week of expenditure of total physical activity was 33.2 MET-hours/week (22.4) and 97 women (11.6%) had a light level of physical activity (< 10), 575 (69.0%) had a moderate level of physical activity (10–50) and 161 women (19.3%) had a vigorous level of physical activity (> 50).

**Table 2:** Physical activity characteristics.

Variables	N (%)
**Walking (m/day)**	
< 500	326 (39.1%)
500–2,000	373 (44.8%)
≥ 2,000	134 (16.1%)
**Flight stairs (n/day)**	
0	302 (36.3%)
1–4	432 (51.9%)
≥ 5	99 (11.9%)
**Moderate recreational activity (hour/week)**	
0	326 (39.1%)
1–2	378 (45.4%)
3–4	94 (11.3%)
≥ 6	35 (4.2%)
**Vigorous recreational activity (hour/week)**	
0	602 (72.3%)
1–2	155 (18.6%)
3–4	50 (6.0%)
≥ 6	26 (3.1%)
**Light household activity (hour/week)**	
0	74 (8.9%)
1–2	415 (49.8%)
3–4	213 (25.6%)
≥ 6	131 (15.7%)
**Heavy household activity (hour/week)**	
0	269 (32.3%)
1–2	353 (42.4%)
3–4	157 (18.8%)
≥ 6	54 (6.5%)
**Missing data**	
Total physical activity (MET-hour/week)	33.2 (± 22.4)
Light physical activity (< 10 MET-hour/week)	97 (11.6%)
Moderate physical activity (between 10 and 50 MET-hour/week)	575 (69.0%)
Vigorous physical activity (> 50 MET-hour/week)	161 (19.3%)

### Demographics and clinical characteristics according to physical activity level

Patients who only completed high-school had a lower total score of physical activity as compared to patients who had a one to two year university degree (*p* = 0.007), and as compared to patients who had ≥ 3-year university degree (p = 0.002). Patients overweight with a BMI between 25 and 30 had a lower total physical activity score than patients who had a normal weight (p = 0.002) and obese patients with a BMI ≥ 30 had also a lower total physical activity score than patients with a normal weight (*p* = 0.001). Smokers (actual or ex-smoker) had a statistically significant higher score of physical activity than non-smokers (*p* = 0.006). Furthermore, characteristics between the three levels of physical activity were statistically significantly different (Table 1) for PS ECOG (*p* < 0.001), age at metastatic diagnosis (*p* < 0.001), skin and pleura metastatic localization (*p* = 0.005), and previous adjuvant chemotherapy (*p* = 0.018). On the other hand, no significant differences were found between the three levels of physical activity for the ER and HER2 status (*p* = 0.283), the median time between diagnosis and metastatic disease (< 24 months versus ≥ 24 months) (*p* = 0.302), the others metastatic localizations and the number of metastatic sites (< 3 sites versus ≥ 3 sites) (*p* = 0.067). For patients with HER2 + metastatic breast cancer, 48 of them (33%) were treated in first line with a double HER-2 blockage; (trastuzumab + pertuzumab: 42 pts; trastuzumab + lapatinib: 6 pts). This proportion was 3/12 (25%), 32/96 (33%), and 13/36 (36%) in the light, moderate and vigorous physical activity subgroup, respectively.

### Physical activity and survival from metastatic relapse

Among the 833 patients who completed the physical activity questionnaire, 475 died and for 96% of them, their cause of death was directly related to their breast cancer. The median length of overall survival for patients who had reported light, moderate and vigorous physical activity was 35.5 months (95% CI 27.8–41.0), 40.9 months (95% CI 36.6–43.2) and 50.4 months (95% CI 40.9–NR), respectively.

**Figure 1:**
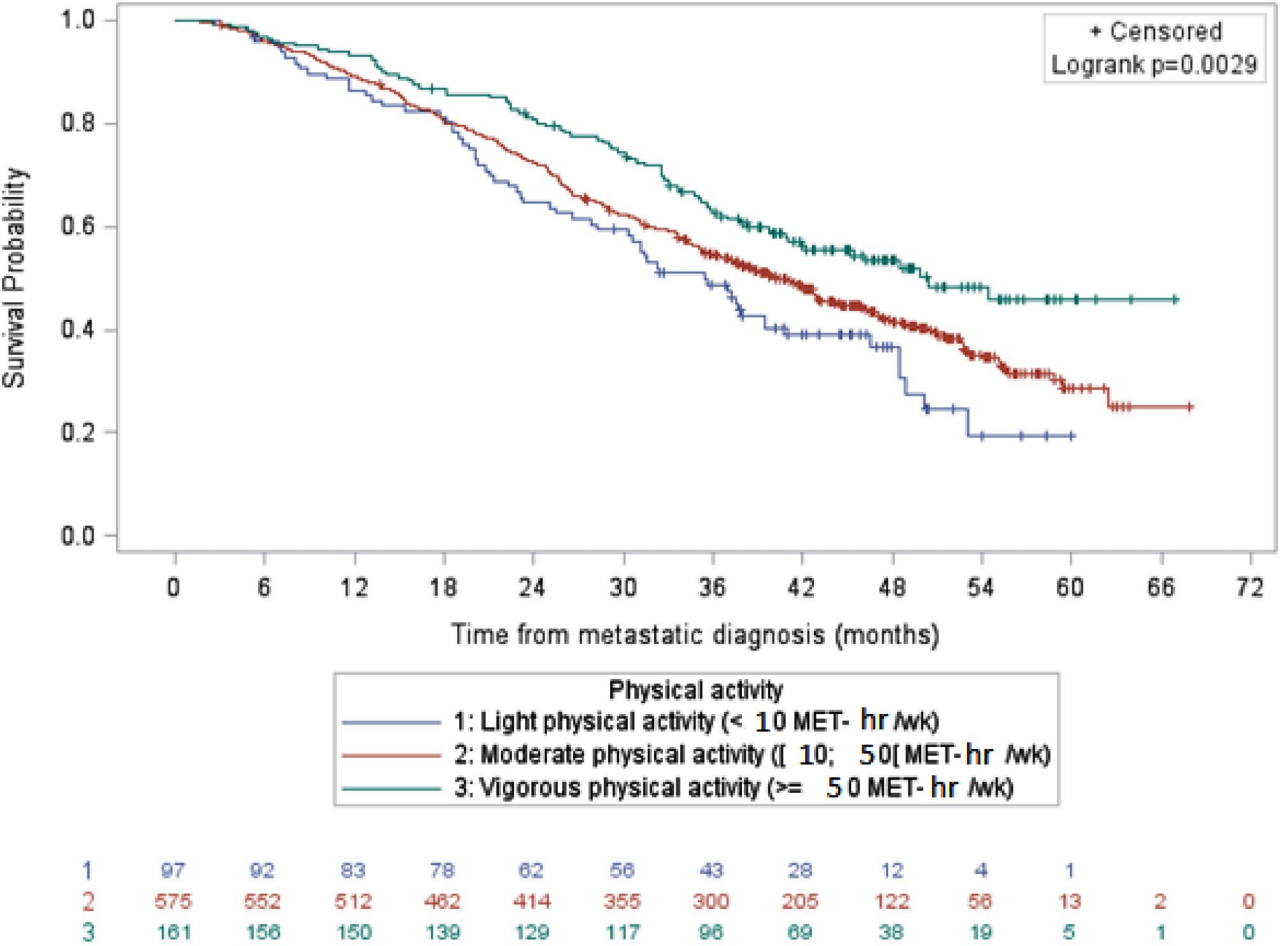
Survival of patients with metastatic breast cancer by physical activity level, data not adjusted (N = 833).

Before adjustment, overall survival was longer for patients with vigorous physical activity as compared to patients with light physical activity (HR 0.57; 95% CI, 0.41–0.79; *p* = 0.0009) (Fig. 1). After adjustment for age at metastatic diagnosis, BMI, tumor type, education, smoker/non-smoker status, stage IV at diagnosis, PS ECOG, number of metastatic localizations, adjuvant chemotherapy and imputations on missing data, moderate and vigorous physical activity levels were not statistically significantly associated with longer survival in the whole population as compared to light physical activity (HR 0.95, 95% CI 0.70–1.29, and HR 0.76, 95% CI 0.53–1.10, respectively).

**Figure 2:**
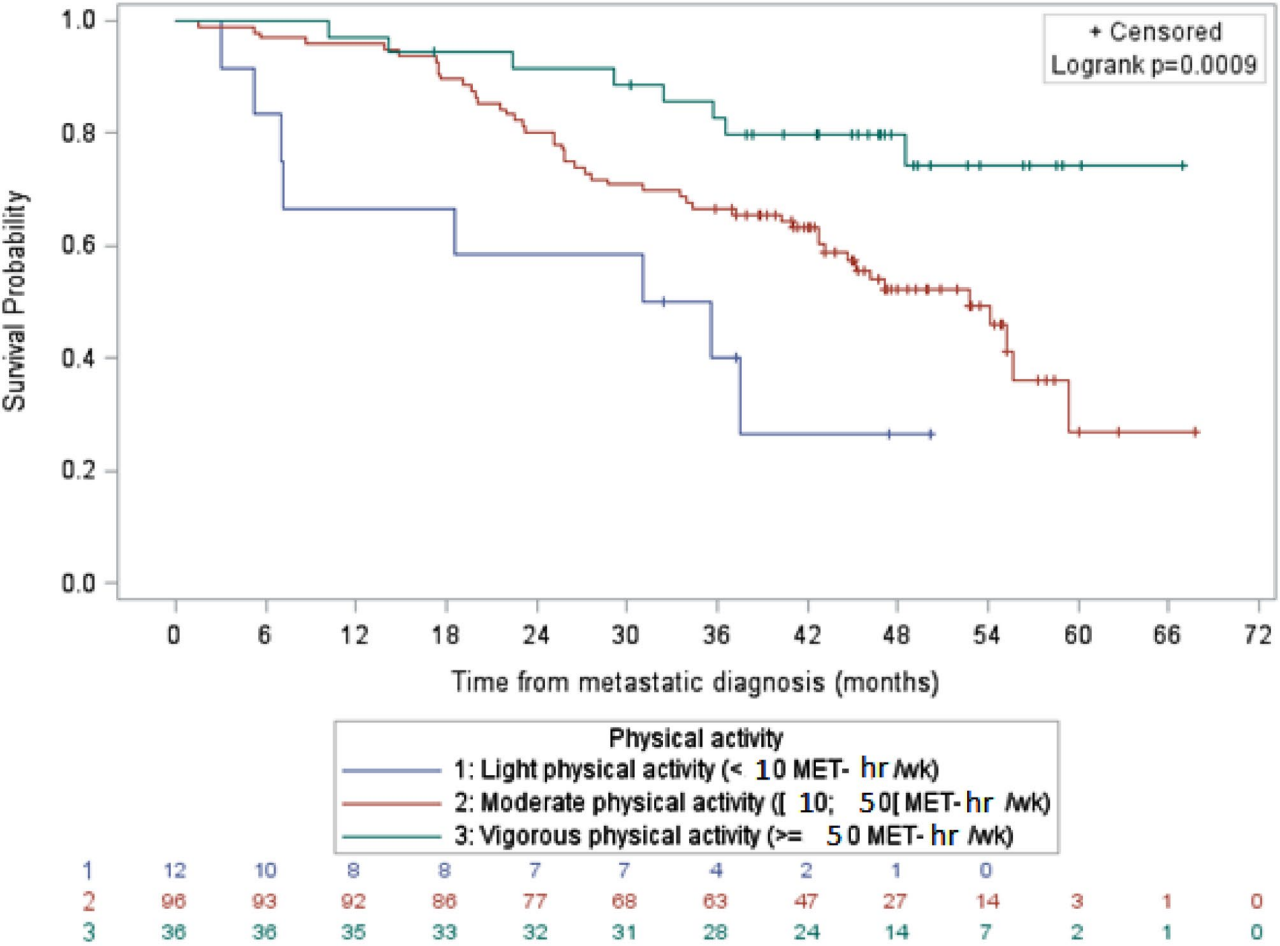
Survival of patients with HER2 positive metastatic breast cancer by physical activity level, data not adjusted (N = 144). It shows overall survival of women with HER2 positive metastatic breast cancer according to the level of physical activity. After adjustment with a Cox regression model on age at metastatic diagnosis, BMI, tumor type, education, smoker/non-smoker status, stage IV at diagnosis, PS ECOG, number of metastatic localizations, adjuvant chemotherapy and imputations on missing data, both vigorous and moderate physical activity were associated with significantly improved overall survival compared to light physical activity level only in the HER2 positive subgroup.

For the HER2 overexpressed metastatic breast cancer subgroup, the median overall survival for patients with light and moderate physical activity was 33.4 months and 52.7 months, respectively (Fig. 2). Median overall survival could not be estimated in the vigorous physical activity level subgroup since more than half of the study population was still alive at the time of analysis These differences remained statistically significant after adjustment on clinical characteristics and treatment, with an HR of 0.38, (95% CI 0.15–0.96) between moderate and light levels of physical activity, and 0.23 (95% CI 0.07–0.70) between vigorous and light levels of physical activity.

**Figure 3:**
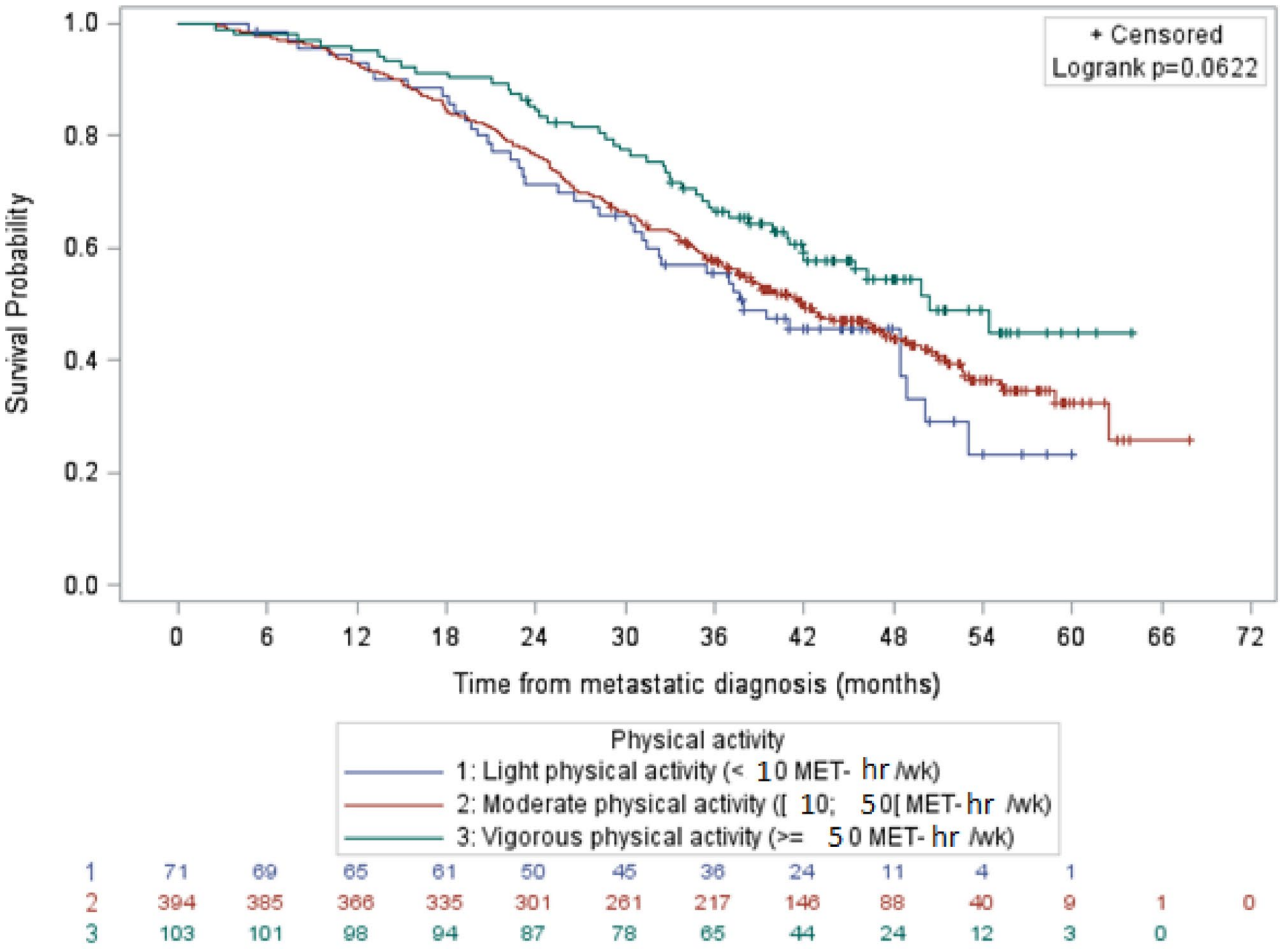
Survival of patients with luminal metastatic breast cancer by physical activity level, data not adjusted (N = 568). It shows overall survival of women with luminal metastatic breast cancer according to the level of physical activity. After adjustment, overall survival according to the level of physical activity was not statistically significant in the Cox model.

For luminal breast cancers, the median lengths of overall survival with light, moderate and vigorous physical activity were 37.9 months, 42.0 months and 50.4 months respectively (Fig. 3). After adjustment, these differences in lengths of survival were not significantly different across the three levels of physical activity.

**Figure 4:**
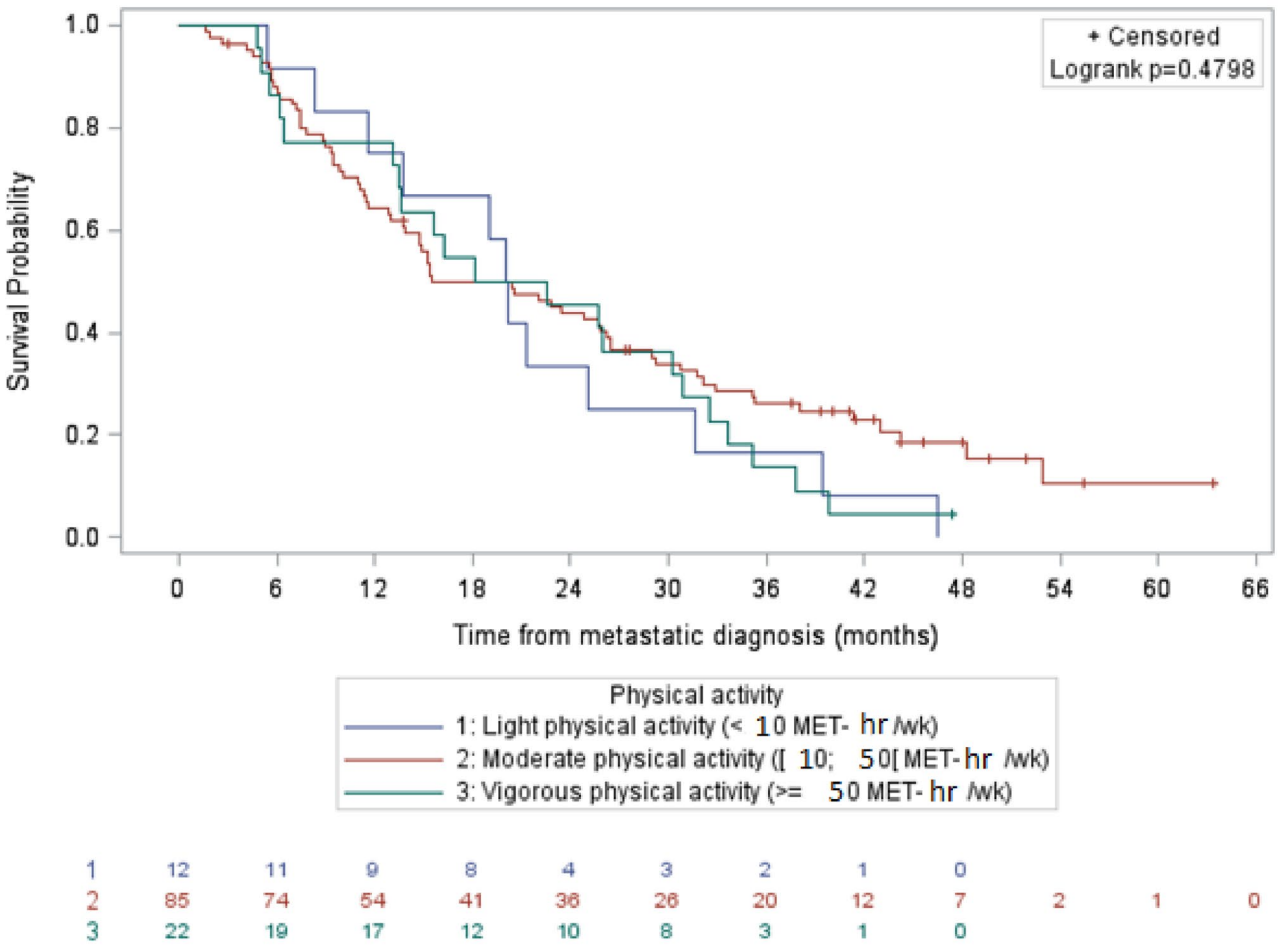
Survival of patients with triple negative metastatic breast cancer by physical activity level, data not adjusted (N = 119). It shows overall survival of women with triple negative metastatic breast cancer according to the level of physical activity. After adjustment, overall survival according to the level of physical activity was not statistically significant in the Cox model.

In triple negative breast cancers, the median lengths of overall survival with light, moderate and vigorous physical activity were 20.2 months (95% CI 8.3–31.6), 15.5 months (95% CI 13.8–26.2) and 20.4 months (95% CI 13.1–30.9), respectively (Fig. 4). These small differences across physical activity levels were not statistically significant in the Cox model.

### Missing data

The StoRM cohort included 1,483 women with a metastatic breast cancer but only 833 participants answered the physical activity questionnaire. We found that the women who responded were statistically significantly younger than the non-responders at diagnosis of primary tumor (*p* < 0.001) and at diagnosis of metastatic disease (*p* < 0.001). There were no other differences between the two groups. No difference in terms of survival between the respondents and non-respondents was found according to tumor subtype.

## Discussion

To our knowledge, this report is the first study to evaluate the impact of physical activity on overall survival in a large prospective cohort of patients with metastatic breast cancer, regardless of histological subtype. We showed that physical activity levels were associated with prognostic factors (lower Performance status, lower age, lower metastases numbers), higher education, lower BMI, and with a better overall survival. After adjustment in a Cox regression model, a moderate or vigorous amounts of physical activity remained statistically significantly associated with better overall survival only for patients with HER2 positive metastatic breast cancer. Patients reporting moderate physical activity or vigorous physical activity had better overall survival than those reporting light physical activity, with an HR of 0.38 (95% CI 0.15–0.96, *p* = 0.04) and an HR of 0.23 (95% CI 0.07–0.70, *p* = 0.01) respectively. On the other hand, survival of luminal and triple negative patients is not affected by physical activity level.

The impact of physical activity for patients with early stage breast cancer has already been demonstrated. Prior to the diagnosis of breast cancer, a physical activity level greater than 3 MET-hours/week versus a physical activity level of less than 3 MET-hours/week reduced overall mortality by 18% (HR 0.82; 95% CI 0.67–0.99)^15,34^. Another meta-analysis confirmed these results: pre-diagnostic physical activity reduced overall mortality by 18% (p < 0.05), specific breast cancer mortality by 27% (p = 0.05) and the risk of carcinogenic events in the breast (recurrence or progression of breast cancer, second cancer) by 28% (HR 0.72, 95% CI 0.56–0.91). In addition, the increase in physical activity level of 5, 10 and 15 MET-hour/week was associated with an overall mortality reduction of 7%, 13% and 19%, respectively^35^. Unlike localized breast cancer for which survival analyses require a large number of subjects and a lengthy follow-up period^15^, the StoRM study yielded statistically significant and clinically relevant results on a smaller number of patients and a shorter follow-up period.

At the metastatic stage, only one single-centered study evaluated the impact of physical activity on survival, but on a small number of patients (n = 103), mostly HR+ (76.7%), and without specific analysis according to histological subgroup^25^. Their study conducted Cox regression analysis on survival by physical activity while controlling for prognostic factors of survival (age, marital status, ER status, treatment, dominant site of metastatic disease spread, depression, and cortisol slope). Their results are consistent with ours: physical activity at baseline was associated with longer survival for women with advanced breast cancer.

Assessing the potential benefit of physical activity on survival in the metastatic setting is challenging. Patients with the worst prognosis are the most likely to have disease-related symptoms and compromised PS that will, in turn, prevent them from having a high level of physical activity. As a consequence, the hypothetical specific contribution of higher physical activity on overall survival may be difficult to establish. To overcome this problem, we were able to conduct Cox regression analysis on the whole population and on the main therapeutic subgroups of patients, i.e. luminal, HER2 positive and triple negative. Interestingly, physical activity levels remained statistically significantly associated with a better overall survival in this multivariate analysis only in the HER2 positive subgroup. One potential confounding factor in this population is first line metastatic treatment since (i) the period of inclusion covered the approval of pertuzumab and (ii) several clinical trials were open to inclusion, patients had heterogeneous treatment with 33% of them having received a double HER2 blockade, known to be associated with better overall survival. As a consequence, we added this information to our multivariate analysis and physical activity levels were still statistically significantly associated with better overall survival. This results could be explained by the fact that this subgroup is the only one for which an aggressive chemotherapy regimen combined with anti-HER2 targeted therapy has clearly shown an improvement in overall survival^6,36,37^. On the other hand, such an improvement from aggressive treatment has never been shown for triple negative or luminal metastatic breast cancer^6,38^*.* Consequently, if moderate/vigorous physical activity levels allow for a better chemotherapy dose-intensity, this benefit will be of most importance only for the patients with HER2-positive disease.

The anti-tumor effect of physical activity can also be explained by different biological mechanisms. Physical activity increases the sensitivity to insulin in healthy or insulin-resistant individuals. Insulin resistance has implications for tumor onset and development^39^. Hyperglycemia increases the glucose availability of tumor cells whose metabolism is mainly glycolytic. Hyperinsulinemia, on the other hand, has an anabolic and antiapoptotic effect^40^. Improved insulin sensitivity reduces the free fraction of IGF-1 that stimulates tumor growth and decreases apoptosis, as well as the free and therefore active fraction of sex hormones (androgens and estrogens)^41^. Physical activity prevents the storage of fatty acids in adipose tissue, thus limiting insulin resistance. Physical activity also reduces visceral fat mass which prevents the conversion of androgens to estrogens by aromatase and estrogen synthesis by adipose tissue cells^42–44^. To confirm the involvement of these different signaling pathways, biomarkers such as IGF-1, estrogen and leptin should be integrated into interventional physical activity studies.

Our study has some limitations. The assessment of physical activity is obtained by questionnaire. The questionnaire used in this study, has already been accepted as a tool in several French studies to measure physical activity and to assess its impact on the risk of developing breast cancer^28,29^. However, the questionnaire used is old and does not adequately represent different types of physical activity such as resistance activities while some aerobic activities, such as stair climbing may be overestimated. Moreover, self-report questionnaires may miss activities and both under- and overestimate the actual levels of physical activity^45,46^. Some patients may overestimate their level of physical activity because they reported the same activity several times in the questionnaire or because they did not know what corresponds to “light”, “moderate” or “vigorous” physical activity. Others may underestimate their level because for them physical activity is equivalent to sport and they do not considered household activities as physical activity. Furthermore, it was not possible to determine the change in physical activity during the course of the metastatic disease and the impact of changes on survival in StoRM because the physical activity questionnaire was only asked at enrollment, and not repeated during follow up. The quantity of missing data may also introduce a bias in our study since women who answered were generally younger, and had a better overall survival than patients who did not respond. Hypothetically, younger women may be more active than older participants and thus may have completed the questionnaire if they felt concerned about this topic. Then, the physical activity variable was categorized into three groups in accordance with WHO physical activity recommendations in order to simplify prevention messages for both clinicians and patients. These findings are recognized as preliminary and further larger-scale studies will be required to confirm these findings.

Nevertheless, given the emerging data on the benefits of physical activity in patients with localized breast cancer and despite the limited data on the effects of a physical activity intervention in metastatic breast cancer, three studies reported the need, desire and ability of these patients to engage in exercise programs^21,24,27,47^. Currently only five intervention studies have focused on the implementation of physical activity in patients with metastatic breast cancer and none in a French cancer center or hospital context^20–24^. Furthermore, intervention studies are needed to determine how and what kind of physical activity may help women with metastatic breast cancer mitigate disease-related symptoms and potentially improve overall survival. For example, the Advanced stage Breast cancer and Lifestyle Exercise (ABLE) single-arm Trial implemented in the Léon Bérard Cancer Center is the first French feasibility study to assess the effects of a six-month physical activity intervention in women with metastatic breast cancer on physical, biological, psychological and clinical parameter^48^. In total, 49 women with metastatic breast cancer were enrolled in the ABLE trial (94% acceptability rate). The proportion of participants achieving the physical activity recommendations (≥ 10.5 MET-hours/week) was not different between inclusion and the end of the study with 34 (69.4%) vs (34 (77.3%) (p = 0.26). This unsupervised physical activity program may encourage patients to maintain a long term physically active lifestyle^49^.

In conclusion, we found that moderate/vigorous levels of physical activity were associated with better overall survival in metastatic breast cancer patients in our exploratory analysis, and that these associations remained statistically significant in multivariate analysis in the HER2 positive subgroup. These results may have important therapeutic implications and justify physical activity intervention studies in metastatic breast cancer.
